# Entomological parameters and population structure at a microgeographic scale of the main Colombian malaria vectors *Anopheles albimanus* and *Anopheles nuneztovari*

**DOI:** 10.1371/journal.pone.0280066

**Published:** 2023-01-06

**Authors:** Mariano Altamiranda-Saavedra, Nelson Naranjo-Díaz, Jan E. Conn, Margarita M. Correa

**Affiliations:** 1 Grupo de Microbiología Molecular, Escuela de Microbiología, Universidad de Antioquia, Medellín, Colombia; 2 Grupo de Investigación Bioforense, Tecnológico de Antioquia, Medellín, Colombia; 3 New York State Department of Health, Wadsworth Center, Albany, NY, United States of America; 4 Department of Biomedical Sciences, School of Public Health, State University of New York-Albany, Albany, NY, United States of America; Fundação Oswaldo Cruz Centro de Pesquisas René Rachou: Fundacao Oswaldo Cruz Instituto Rene Rachou, BRAZIL

## Abstract

Population subdivision among several neotropical malaria vectors has been widely evaluated; however, few studies have analyzed population variation at a microgeographic scale, wherein local environmental variables may lead to population differentiation. The aim of the present study was to evaluate the genetic and geometric morphometric structure of *Anopheles nuneztovari* and *Anopheles albimanus* in endemic localities of northwestern Colombia. Genetic and phenetic structures were evaluated using microsatellites markers and wing geometric morphometrics, respectively. In addition, entomological indices of importance in transmission were calculated. Results showed that the main biting peaks of *Anopheles nuneztovari* were between 20:00 and 22:00, whereas *Anopheles albimanus* exhibited more variation in biting times among localities. Infection in *An*. *nuneztovari* by *Plasmodium* spp. (IR: 4.35%) and the annual entomological inoculation rate (30.31), indicated high vector exposure and local transmission risk. We did not detect *Plasmodium*-infected *An*. *albimanus* in this study. In general, low genetic and phenetic subdivision among the populations of both vectors was detected using a combination of phenotypic, genetic and environmental data. The results indicated high regional gene flow, although local environmental characteristics may be influencing the wing conformation differentiation and behavioral variation observed in *An*. *albimanus*. Furthermore, the population subdivision detected by microsatellite markers for both species by Bayesian genetic analysis provides a more accurate picture of the current genetic structure in comparison to previous studies. Finally, the biting behavior variation observed for both vectors among localities suggests the need for continuous malaria vector surveys covering the endemic region to implement the most effective integrated local control interventions.

## Introduction

Malaria remains one of the most important public health problems worldwide. The *Plasmodium* parasites that cause the disease are transmitted to humans by the female mosquitoes of approximately 40 species of *Anopheles* [1]. Colombia is third in the number of malaria cases in the Americas [2], and the number of cases/year in the past five years has fluctuated between 50,000–80,000 [3–7]. Currently, the most malaria endemic regions are the Pacific (PAC) in western Colombia (49.2%), and the Urabá-Bajo Cauca-Alto Sinú (UCS), in the northwest (18.7%) [3]. During the study period (2013–2014), the number of malaria cases registered in UCS were 22,889 (2013) and 8,620 (2014), corresponding to 43.84% and 20.97% of the cases in the country, respectively [8, 9]. The main malaria vectors in Colombia are *Anopheles darlingi*, *Anopheles nuneztovari* and *Anopheles albimanus* [10, 11]. Vector control is one of the main strategies to decrease malaria incidence [1]; thus, knowledge of vector biology remains essential to reduce malaria transmission. It is known that anthropogenic environmental alterations and insecticide selection pressures affect vector population dynamics, because these factors may increase the abundance of anthropogenic *Anopheles* species and the appearance of insecticide resistant species, which affect malaria risk parameters [12–14]. Hence, genetic population structure and entomological parameters should be regularly monitored to evaluate changes in transmission risk to implement Integrated Vector Management programs [15]; such information will allow, for example, the identification of season(s) when vector control interventions should be intensified.

Population structure studies of the neotropical malaria vectors *An*. *nuneztovari* and *An*. *albimanus* have identified population subdivision across their distributions [16, 17]. Regarding *An*. *nuneztovari*, several lineages were identified among South American populations [18], and a new species of the Nuneztovari Complex, lineage III, has been identified east of the Amazon region [19]. In Colombia, genetic differentiation and population subdivision was detected between *An*. *nuneztovari* populations from the west-northwest and the east-northeast, attributed to physical barriers, geographic distance and ecological variation on both sides of the Andes [20]. However, a study using *COI* and the *white* gene found high gene flow and the existence of a single taxon in *An*. *nuneztovari* from UCS, northwest Colombia [21]. Various studies support the status of *An*. *albimanus* as a single taxon in Central and South America [22, 23], and genetic structure was identified between Central and South American populations [24]. In Colombia, *An*. *albimanus* is mainly distributed on the Atlantic and Pacific coasts [11, 25], where the populations show little genetic differentiation [26, 27]. The combination of genetic and morphometric data found low genetic structure and high gene flow, confirming *An*. *albimanus* as a metapopulation [26]. Both species have been detected infected with *Plasmodium* spp., *An*. *nuneztovari* in UCS-northwest and in PAC-west Colombia, and *An*. *albimanus* in PAC [25, 28, 29].

Previous studies in the Colombian malaria endemic UCS suggest that anthropogenic activities such as mining, livestock production, timber extraction, small scale agriculture and wood exploitation favor the presence of the main vectors *An*. *albimanus* and *An*. *nuneztovari*. These activities promote landscapes that impact key mosquito parameters, such as mosquito species composition, vector abundance, biting behavior and natural parasite infection, which determine malaria transmission risk at the local and regional levels [12, 30, 31]. The present study evaluates geometric morphometrics and genetic population variation in addition to entomological parameters for *An*. *nuneztovari* and *An*. *albimanus* at a microgeographic scale in the UCS region of northwest Colombia. These analyses may provide important information on the factors determining mosquito population variation that can influence vector transmission dynamics.

## Methods

### Study area and sample collection

**Fig 1:**
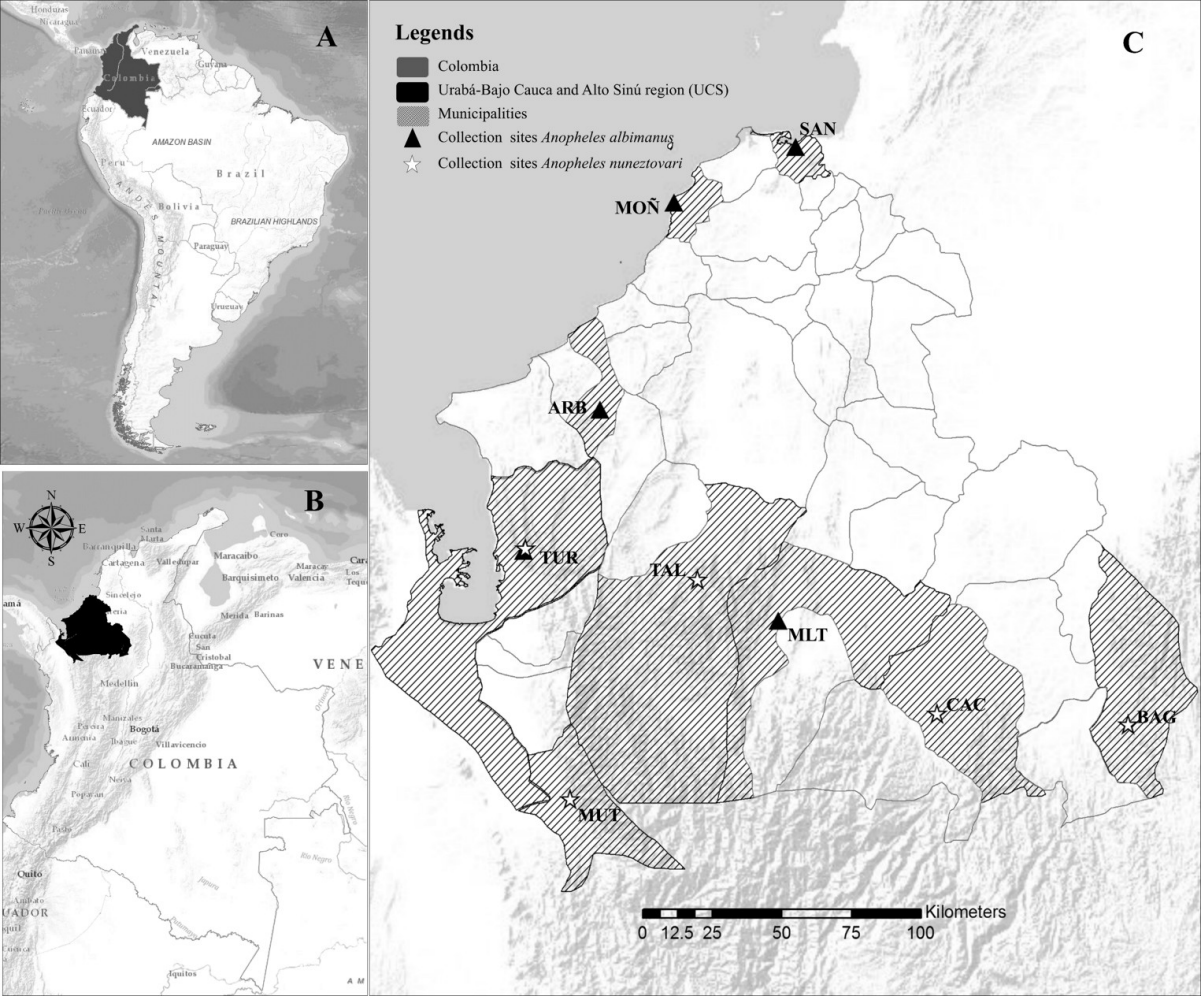
Collection sites. A. Map shows the location of Colombia in relation to South America. B. The malaria endemic region Urabá-Bajo Cauca-Alto Sinú in northwest Colombia. C. Municipalities where specimens were collected: BAG: El Bagre, CAC: Cáceres, MUT: Mutatá, TAL: Tierralta, TUR: Turbo, MOÑ: Moñitos, ARB: Arboletes, SAN: San Antero and MLT: Montelíbano. Stars: municipalities where *An*. *nuneztovari* specimens were collected. Triangles: municipalities for *An*. *albimanus*. Map created by the authors in ArcGIS, Release 10.2. Redlands, CA.

*Anopheles* specimens were collected from two localities in each of five municipalities, six sites per locality, in the malaria endemic region Urabá-Bajo Cauca and Alto Sinú (UCS) in northwest Colombia (Table 1, Fig 1). Selection of the localities and collection times was based on reports of malaria transmission and safety considerations. Collections were performed from September 2013 to October 2014, during three nights per locality from 18:00–24:00 h, in open areas of the house and in the peri-domestic area using protected humans as an attractant (Informed consent agreement and collection protocol reviewed and approved by a University of Antioquia Institutional Review Board -Bioethics Committee, Facultad Nacional de Salud Pública-Universidad de Antioquia, Acta 063). A written informed consent was obtained from all individuals participating in the collections. The specimens were identified using a morphological key [10] and species assignment was confirmed by PCR-RFLP-ITS2 [32–34] and *COI* barcode gene sequencing [35, 36].

**Table 1:** Data on *Anopheles nuneztovari* and *Anopheles albimanus* specimens from Urabá-Bajo Cauca and Alto Sinú, northwest Colombia, analyzed by microsatellites.

Municipality/Localities	Longitude	Latitude	m.a.s.l.	Relevant human activities (Field observations)	Ter Ecore*	Year / month of collection	Species	MS *n*	SMP *n*
**El Bagre**						2013/September	*An*. *nuneztovari*	29	30
La Lucha	-74.719611	7.594389	130	Artisanal mining	MU mf				
Villa Grande	-74.704611	7.533361	102	Cattle raising	MU mf				
**Mutata**						2014/February	*An*. *nuneztovari*	28	30
Bejuquillo	-76.50642	7.367030	137	Pisciculture	CD mf				
La Secreta	-76.586139	7.308778	63	Agriculture	CD mf				
**Turbo**						2014/November	*An*. *nuneztovari*	30	27
La Playona	-76.656028	8.134028	23	Extensive cattle raising	CD mf				
Camerun	-76.727972	8.152056	8	Plantain and banana crops	CD mf				
**Caceres**						2014/May	*An*. *nuneztovari*	24	30
Asturias	-75.319417	7.623250	92	Cattle ranching	MU mf				
Campanario	-75.230139	7.582472	196	Agriculture	MU mf				
**Tierralta**						2014/October	*An*. *nuneztovari*	28	30
Tuistuis	-76.094294	8.046328	132	Agriculture and fishing	NA mf				
Santa Ana	-76.175222	8.026167	142	Agriculture	NA mf				
**Arboletes**						2013/March	*An*. *albimanus*	28	26
Naranjita	-76.333583	8.530694	70	Agriculture	MU mf				
La Arenosa	-76.412583	8.567472	72	Agriculture and cattle ranching	MU mf				
**Moñitos**						2013/November	*An*. *albimanus*	29	30
Broqueles	-76.151361	9.216583	20	Agriculture and pisciculture	MU mf				
Rio Cedro	-76.157667	9.168583	4	Agriculture and tourism	MU mf				
**San Antero**						2013/November	*An*. *albimanus*	30	30
Bahia Cispata	-75.774694	9.395638	2	Tourism	MU mf				
Tijereta	-75.788916	9.293583	48	Agriculture and cattle ranching	MU mf				
**Turbo**						2014/November	*An*. *albimanus*	30	30
La Playona	-76.656028	8.134028	23	Extensive cattle raising	CD mf				
Camerun	-76.727972	8.152056	8	Plantain and banana crops	CD mf				
**Montelíbano**						2014/July	*An*. *albimanus*	24	30
Puerto Anchica	-75.851056	7.869861	105	Agriculture and cattle ranching	NA mf				
Puerto Nuevo	-75.833306	7.915333	129	Agriculture and fishing	NA mf				

Coordinate system: UTM-WGS84. *Terrestrial Ecoregions [37], MUmf: Magdalena-Urabá moist forests, CDmf: Chocó-Darién moist forests, NAmf: Northwestern Andean montane forests, MS *n* = Specimens analyzed by microsatellites; SMP *n* = Specimens analyzed by geometric morphometrics; m.a.s.l.: meters above sea level.

### Analysis of genetic data

Given the geographical proximity among localities from the same municipality, specimens were grouped and analyzed by municipality for the genetic analysis. Inter and intra-population genetic diversity was estimated for 139 *An*. *nuneztovari* specimens and 141 *An*. *albimanus* (Table 1). For *An*. *nuneztovari*, 11 microsatellite (MS) loci were analyzed (Anu3, Anu7, Anu11, Anu12, Anu10, Anu1, Anu2, Anu9, Anu4, Anu6, Anu8) [19], (S1 Table) and nine loci for *An*. *albimanus* (107, 113,124, 128, 2–14, 117, 6–41,78, 2–25) [24, 38] (S2 Table). The amplification of target sequences was performed by a sequence service facility at Universidad de Antioquia, where multiplex PCRs were performed (95°C 5 min; 28 cycles of 94°C for 30 sec, 56°C for 30 sec, 72°C for 30 sec; final ext. 72°C for 30 min); the products were run by capillary electrophoresis using an Applied Biosystems 3130 Genetic Analyzer and the allelic data were edited using GeneMapper v. 3.2. Allele data from a Microsoft Excel database were converted to the appropriate file formats for analysis by specific programs using Convert v.1.31 [39]. The presence of null alleles was evaluated with Micro-Checker v. 2.2.3 [40]. Genetic population structure at the population and individual levels was analyzed using a grouping method based on both transient Hardy–Weinberg disequilibrium (HWD) and linkage disequilibrium (LD) caused by admixture between populations, in Structure v.2.3.4 [41]. Based on allele diversity data, individuals with unique alleles were grouped together into assumed populations (*K*) which is pre-determined [42]. The *K* value with the maximum posterior probability Pr (X|K) was retained and assumed to be the most probable number of clusters in that population. Twenty independent runs were performed for each value of *K* (*K* = 1 to 10) with a burnin period of 500,000 iterations and 2 million steps for Monte Carlo Markov Chain (MCMC) replications run using Structure Harvester v.0.56.3 [43]. A total of 25 files with optimal *K* were averaged using the Large *K* Greedy method implemented in Clumpp v. 1.1.2 [44]. A Cavalli-Sforza chord distance and Neighbor Joining (NJ) consensus tree representing genetic differentiation among populations was created using Populations v. 1.2.31 [45] with 1000 bootstrap replicates [46]. Population differences defined by Structure were visualized using a Factorial Correspondence Analysis (FCA) in Genetix v. 4.05.2 [47].

The number of alleles (*Na*), expected heterozygosity (*He*), observed heterozygosity (*Ho*), allele richness (*Rs*), number of migrants per generation (*Nm*), inbreeding index (*F*_*IS*_), fixation index (*F*_*ST*_) and balance of Hardy-Weinberg (HWE) were calculated for each MS loci using Arlequin software v. 3.5 [48]. The number of private alleles for all loci was obtained using Convert v. 1.31 [39]. Molecular analysis of variance (AMOVA) was used to examine within and among populations variation in Arlequin; the allelic frequencies were used to estimate Euclidean distances at different levels of genetic structure, with 10,000 non-parametric permutations [48].

### Morphometric data analysis

Wing size and conformation variation were evaluated by geometric morphometric analysis. The right wings of 147 *An*. *nuneztovari* specimens (27–30 per population) and 146 *An*. *albimanus* (26–30 per population) (Table 1), were mounted on microscope slides with coverslips and photographed using a digital camera (Moticam® 2500), attached to an Olympus ® Sz61 stereomicroscope. Right wing images were used for morphometric analysis and a set of 13 reference measurements were considered in the analysis [26]. To estimate variation of wing size and conformation, raw data coordinates were overlaid using a Procrustes full fitting procedure that eliminates variation due to scale, position and orientation of datum settings [49]. Wing size variation was examined using the centroid size (CS), defined as the square root of the sum of the square distances of a set of reference points from the centroid [50]. CS variation was tested using a Mann-Whitney test after Bonferroni correction [51].

Wing conformation among populations was compared by means of a disparity analysis, using 10,000 random permutation tests. To calculate the differences in wing conformation among populations, allometry-free variables were used as input for a Canonical Variates Analysis (CVA). To obtain Procrustes and Mahalanobis distances, the CVA analysis was used to determine whether the five geographic populations of each species could be statistically distinguished based on the relative deformation matrix [26, 52]. Means of the wing conformation for specimens of the five geographic populations were plotted along the two axes of canonical variation based on the Procrustes distance matrix [26, 52]. Reference point digitization, morphometric analysis and graphical results were performed using various modules of the Clic package [53]. The Past program was used for wing size comparisons [54].

### Relationship among phenotypic, genotypic and environmental data

Population structure based on allele frequency variation of georeferenced individual genotypes and inference of the number of populations and spatial location of genetic discontinuities between those populations was evaluated by a Bayesian clustering algorithm in Geneland 4.0.3 [55], implemented in R software v. 3.3.2 (R Development Core Team 2008). To estimate the optimal number of subpopulations, based on the spatial location of the sampling sites, a set of data was analyzed using combinations of the phenotypic (P), genetic (G) and spatial (S) matrices, as follows: 1) phenotypic data under the spatial model; 2) genetic data under the spatial model; and 3) phenotypic and genetic data under the spatial model [24]. Potential spatial distribution models (Spatial distribution model-SDM) were generated in Maxent v. 3.3.3 [56], using as input the layers of the Normalized Difference Vegetation Index (NDVI) and *An*. *nuneztovari* and *An*. *albimanus* occurrence records throughout the endemic region, to generate an environmental resistance matrix in Circuitscape v. 3.5.8 [57]. Finally, the relationship between environmental heterogeneity with geographic and with genetic distances, and phenotypic variation of the population, was evaluated using the partial Mantel test [58]. The matrices of genetic structure (*F*_*ST*_), phenotypic differentiation (Mahalanobis distances), environmental or cost distances and geographical distances were compared using R v. 3.3.2.

### Entomological parameters and entomological inoculation rate for *Plasmodium*

Mosquito parasite infection was tested in the pools of five mosquito heads and thoraxes by an enzyme-linked immunosorbent assay (ELISA) using monoclonal antibodies against *Plasmodium falciparum*, *Plasmodium vivax* VK247 and VK210. The positive pools were confirmed by a second ELISA and a nested PCR performed with DNA extracted from the mosquito abdomen and *Plasmodium* specific primers [29, 59]. The entomological parameters were estimated by locality and included, biting behavior that was calculated as the number of mosquitoes of each species collected by hour and locality. Human Biting Rate (HBR), estimated as the average number of *Anopheles* females collected per hour and per average number of collectors, expressed as the number of bites per person, per night (b.p.n) [60]. Infection rate (IR) was calculated as the percentage of *Plasmodium* positive mosquitoes out of the total number of mosquitoes analyzed, by species and locality. The annual entomological inoculation rate (EIR) per site that corresponds to the number of infective bites that a person may receive in one year [29].

## Results

### Genetic diversity and population genetic structure

**Fig 2:**
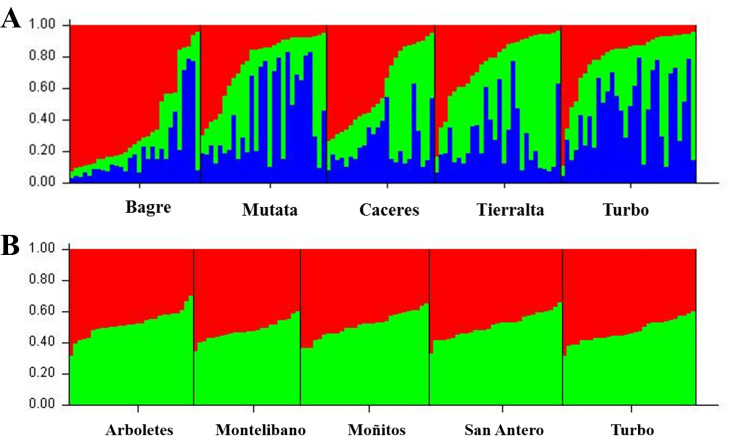
Population structure of (**A**) *Anopheles nuneztovari* (**B**) *Anopheles albimanus*, from five geographic locations in Urabá-Bajo Cauca and Alto Sinú. Each bar represents an individual and the proportion of colored bars represents allocation to subpopulation.

Genetic diversity analyses for *An*. *nuneztovari* showed that the ANU11 locus was not polymorphic and was thus excluded from population analyses. For *An*. *albimanus* all loci were polymorphic. In *An*. *nuneztovari*, the expected mean loci heterozygosity (*He*) ranged from 0.613 in El Bagre to 0.674 in Mutatá and for *An*. *albimanus* from 0.802 in Turbo to 0.840 in Arboletes (Table 2); also, the number of alleles, observed heterozygosity and inbreeding coefficient differs between both species but not as much between localities for each species (Table 2). The *F*_*ST*_ and *Nm* values indicated low genetic differentiation and high gene flow among *An*. *nuneztovari* populations in the UCS region. The average *Nm* was high, with the highest value between Cáceres and Tierralta (64.5) (S3 Table). Bayesian clustering analysis in Structure revealed three subpopulations of genetically related individuals (*K* = 3). There was no particular association among these subpopulations and geographic location (Fig 2A). Similarly, *F*_*ST*_ and *Nm* values for *An*. *albimanus* in UCS showed low genetic differentiation and high gene flow among populations. The average *Nm* was high, with the highest value detected between Moñitos and San Antero (526.4) (S4 Table). The genetic structure analysis revealed two subpopulations of genetically related individuals (*K* = 2); however, there was no association among these subpopulations and geographic location (Fig 2B).

**Table 2:** Genetic diversity statistics for *Anopheles nuneztovari* and *Anopheles albimanus* from Urabá-Bajo Cauca and Alto Sinú, northwest Colombia.

Statistics	*n*	*Na*	*He*	*Ho*	*F*_*IS*_
***An*. *nuneztovari***					
**El Bagre**	29	5.400	0.613	0.417	0.302
**Mutatá**	28	5.800	0.674	0.628	0.135
**Cáceres**	24	4.700	0.614	0.550	0.179
**Tierralta**	28	5.000	0.637	0.589	0.207
**Turbo**	30	5.500	0.628	0.558	0.209
**Average of all populations**		5.280	0.633	0.548	0.206
***An*. *albimanus***					
**Arboletes**	28	11.222	0.840	0.761	0.089
**Montelibano**	24	9.000	0.810	0.721	0.105
**Moñitos**	29	11.333	0.836	0.730	0.124
**San Antero**	30	10.778	0.815	0.715	0.111
**Turbo**	30	10.444	0.802	0.727	0.083
**Average statistics for all populations**		10.556	0.820	0.731	0.102

*n*: sample size, *Na*: number of alleles, *He*: expected heterozygosity, *Ho*: observed heterozygosity, *F*_*IS*_: coefficient of inbreeding.

### Population phenotypic variation

**Fig 3:**
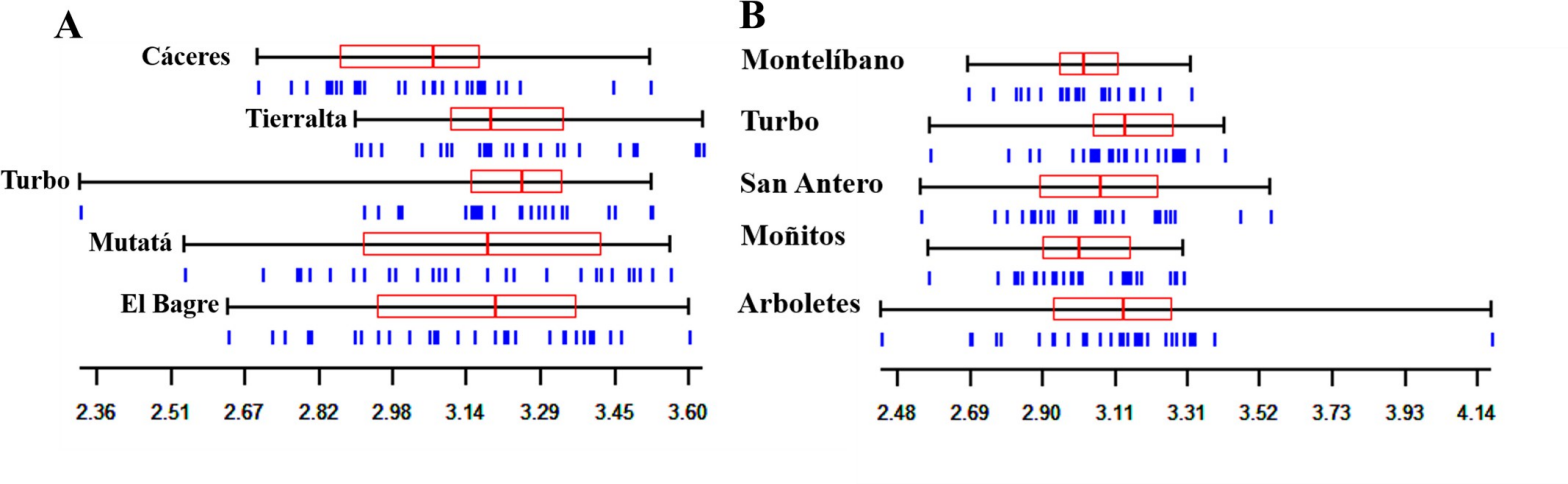
Wing centroid size for (**A**) *Anopheles nuneztovari* (**B**) *Anopheles albimanus* from five municipalities of Urabá-Bajo Cauca-Alto Sinú. The blue vertical lines represent individual specimens. Each box denotes the median as a vertical line. Scale in mm.

Geometric morphometry analysis of 60 *An*. *nuneztovari* wings showed good repeatability in the x, y coordinates (*R* = 0.953), centroid size (*R* = 0.899) and relative deformations (*R* = 0.937). The Mann-Whitney test revealed significant differences for various centroid sizes in the paired comparisons. The analysis of 60 *An*. *albimanus* wings also showed good repeatability in the x, y coordinates (*R* = 0.981), centroid size (*R* = 0.992) and relative deformations (*R* = 0.907) (Fig 3A, S5 Table). Paired comparisons using the Mann-Whitney test showed significant differences for various centroid sizes (Fig 3B, S6 Table), mainly for the Arboletes population compared to the four others.

**Fig 4:**
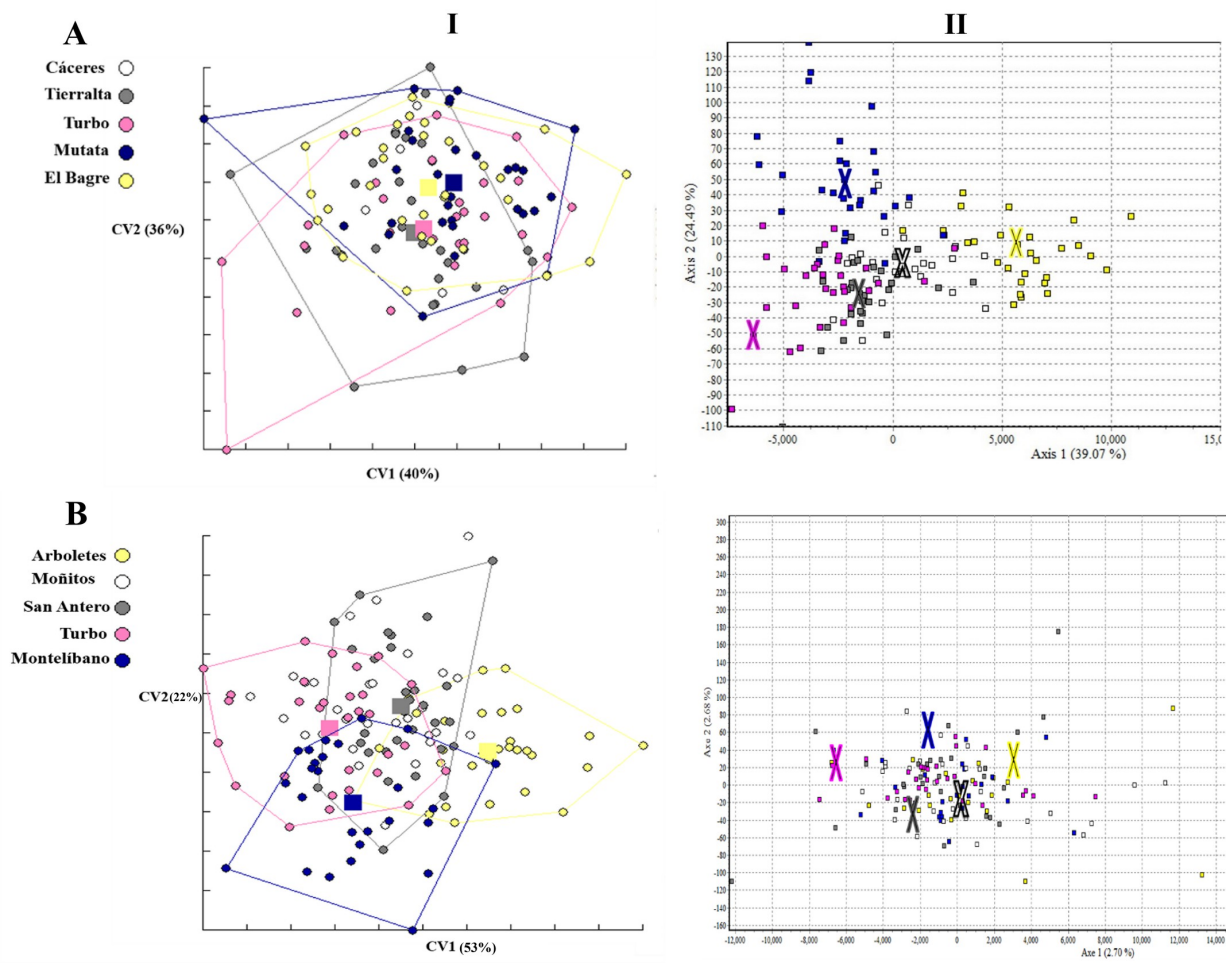
Metric disparity analysis of wing conformation and genetic differences for (**A**) *Anopheles nuneztovari* (**B**) *Anopheles albimanus*. **(I)** Analysis of canonical variation for wing conformation. **(II)** Factorial correspondence analysis based on allele differences, from five populations of Urabá-Bajo Cauca-Alto Sinú. Different colored squares in figures AI and BI, and the Xs in figures BI and BII, represent the means of each population.

The metric disparity analysis for *An*. *nuneztovari* wing conformation showed significant differences between the following comparisons: Cáceres and Mutatá (*p* = 0.0145), Cáceres vs El Bagre (*p* = 0.0007), Tierralta and Mutatá (*p* = 0.0433), Tierralta and El Bagre (*p* = 0.0140) and Turbo and El Bagre (*p* = 0.0152). Discrimination among populations in the morphospace showed that the first two CV axes represented 76% of the total variation of the data (Fig 4AI). Genetic differences among populations analyzed by FCA showed that axes 1 and 2 explained 39.07 and 24.49%, respectively (Fig 4AII); this analysis showed a slight separation of the El Bagre population. For *An*. *albimanus* the wing shape metric disparity analysis revealed no significant differences between the paired comparisons. The discriminant analysis among populations in the morphospace showed that the first two CV axes represented 75% of the total variation of the data (Fig 4BI). The genetic differences among *An*. *albimanus* populations analyzed by FCA showed that axes 1 and 2 explained 2.70 and 2.68% respectively (Fig 4BII). The phenotypic discriminate analysis showed a slight separation of the Arboletes population, observed to a lesser degree in the genetic analysis.

### Combination of phenotypic, genetic and environmental data

**Fig 5:**
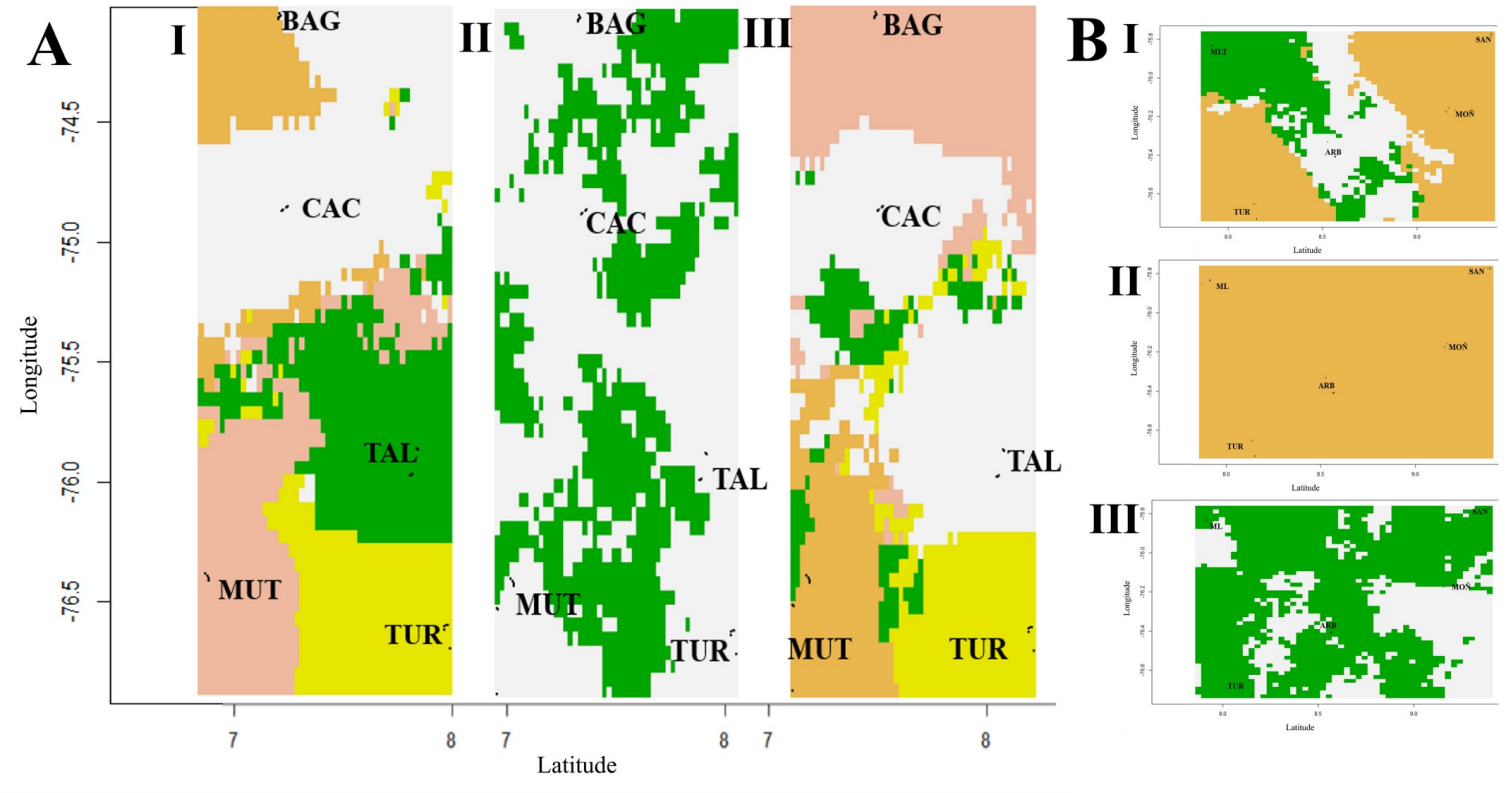
Bayesian clustering results from GENELAND for (**A**) *Anopheles nuneztovari* (**B**) *Anopheles albimanus* collected in five populations of Urabá-Bajo Cauca and Alto Sinú. The colors represent the subpopulations. **I.** Genetic data. **II.** Phenotypic data. **III.** Genetic and phenotypic and data. MOÑ: Moñitos, ARB: Arboletes, SAN: San Antero, MLT: Montelíbano. TUR: Turbo. BAG: El Bagre, CAC: Cáceres, MUT: Mutatá and TAL: Tierralta.

Bayesian analysis of genetic data in GENELAND, which considers the spatial distribution model, showed the presence of five *An*. *nuneztovari* genetic subpopulations (Fig 5AI). However, the same analysis with only the phenotypic data showed that there are two subpopulations (Fig 5AII); the phenotypic-genetic data corroborated the existence of five subpopulations but with a different geographic distribution (Fig 5AIII). A significant correlation was found between genetic vs. geographic distances by the Mantel test (S7 Table). Three *An*. *albimanus* genetic subpopulations were identified by Bayesian analysis (Fig 5BI). However, the same analysis with only phenotypic data showed only one subpopulation (Fig 5BII), while the phenotypic and genetic data revealed two subpopulations (Fig 5BIII). A Mantel test showed no significant correlations among any of the distance matrices (S8 Table).

### Biting behavior

**Fig 6:**
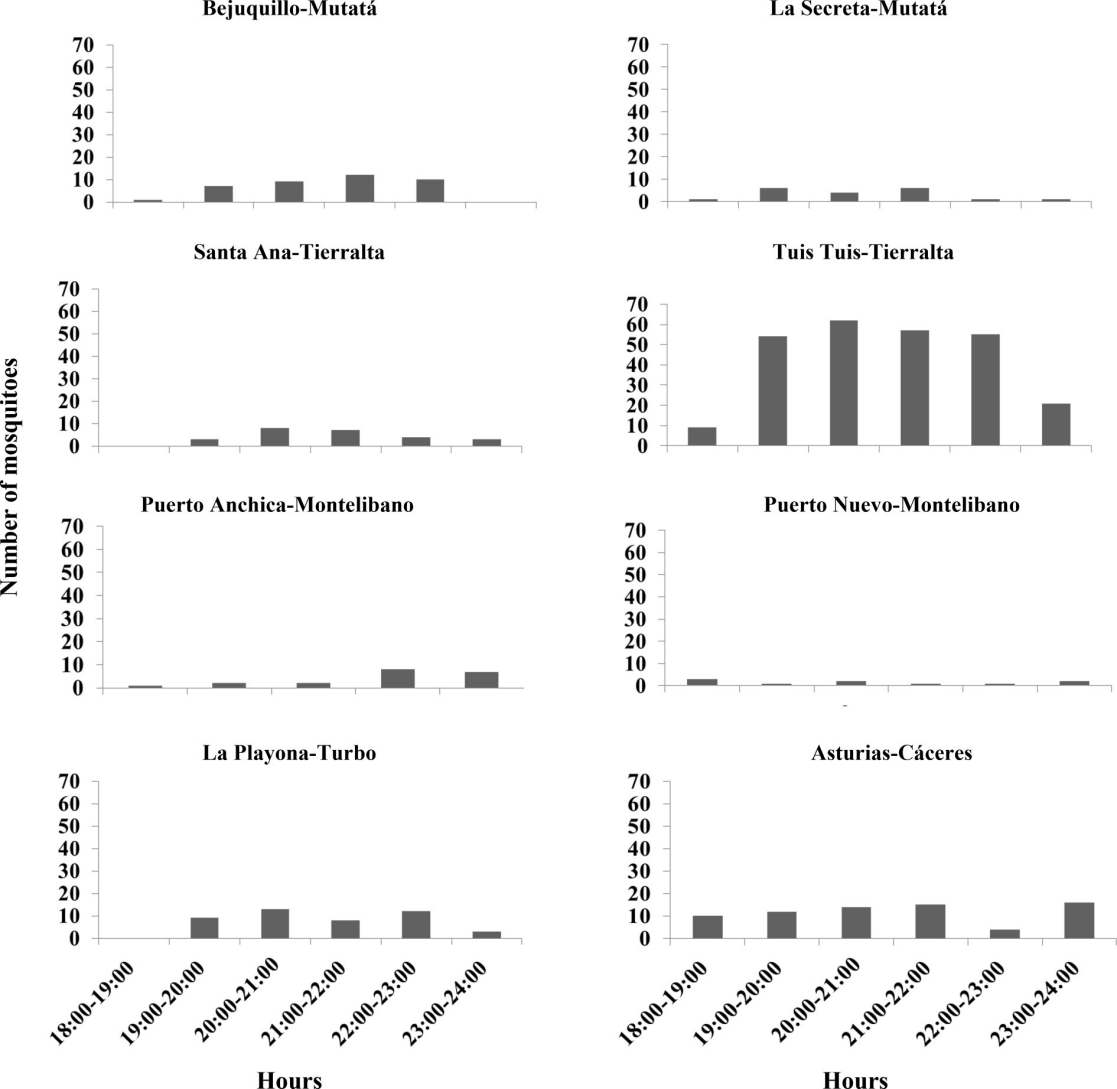
Biting behavior in *An*. *nuneztovari*. Y-axis represent the number of mosquitoes collected and X-axis the hours of collection.

**Fig 7:**
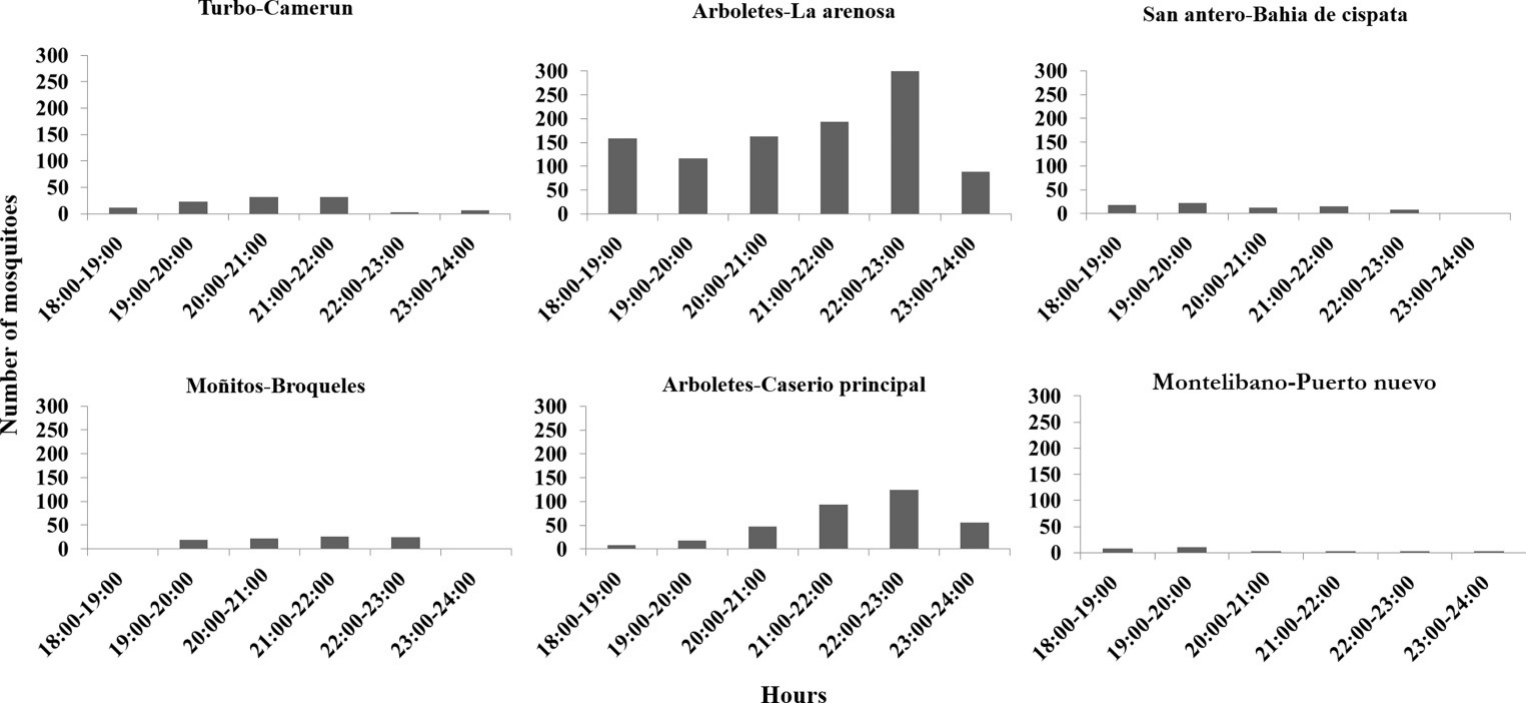
Biting behavior in *An*. *albimanus*. Y-axis represent the number of mosquitoes collected and X-axis the hours of collection.

Intra- and interspecific differences were observed in *An*. *nuneztovari* (Fig 6) and *An*. *albimanus* biting behavior (Fig 7). For *An*. *nuneztovari*, the main biting peaks were between 20:00h and 22:00 h, however, differences were observed in the Montelibano localities with a main peak at 18:00 h in Puerto Nuevo and at 21:00 h in Puerto Anchica. A late peak at 23:00 h was observed in Asturias, Cáceres municipality (Fig 6). The localities Camerún in Turbo and Rio Cedro in Moñitos were not included in the biting behavior analysis because of low mosquito density. Variation in biting times was observed for *An*. *albimanus* among the localities, however, two main biting peaks were evident: one in the early evening (18:00–20:00) h in Bahia Cispatá and Puerto Nuevo and another from 20:00–23:00 h in Camerún-Turbo, Broqueles-Moñitos, La Arenosa and the main settlement of Arboletes (Fig 7).

### Entomological parameters

The human biting rates (HBR) in most localities were relatively homogeneous with rates up to 6 bites per person per night (b.p.n) for *An*. *nuneztovari* and 9 b.p.n. for *An*. *albimanus* (Table 3). The highest HBRs for both species were registered in Asturias-Caceres for *An*. *nuneztovari* (5.91 b.p.n) and in La Arenosa-Arboletes for *An*. *albimanus* (53.6 b.p.n; Table 3). The lowest rates were detected in Camerún-Turbo (0.25 b.p.n.) for *An*. *nuneztovari* and Santa Ana-Tierralta (0.33 b.p.n.) for *An*. *albimanus*. The infection rate was of 4.35%, given the detection of one *An*. *nuneztovari* specimen infected by *Plasmodium* spp., in Puerto Anchica-Montelibano, for an annual EIR of 30.31 infective bites per year (Table 3).

**Table 3:** Human biting rate, infection rate and entomological inoculation rate for *An*. *nuneztovari* and *An*. *albimanus*.

Municipality	Locality	*An*. *nuneztovari* abundance	*An*. *albimanus* abundance	Entomological *An*. *nuneztovari* HBR (IR, EIR)	Parameters *An*. *albimanus* HBR	*An*. *nuneztovari* tested for *Plasmodium* infection *(*n*)	*An*. *albimanus* tested for *Plasmodium* infection * (*n*)
Mutatá	Bejuquillo	39	8	3.25	0.66	65/24	8/8
	La Secreta	19	-	1.58	-		
Turbo	La Playona	45	8	3.75	0.66	50/18	119/81
	Camerún	3	106	0.25	8.83		
Cáceres	Asturias	71	-	5.91	-	75/35	
Tierralta	Santa Ana	25	4	1.38	0.33	285	
	Tuis-Tuis	258	-	19	-		
Arboletes	La Arenosa	-	1019	-	53.6		1365/477
	Naranjita	-	347	-	28.9		
Moñitos	Broqueles	-	92	-	7.6	9	281/146
	Rio Cedro	6	11	0.5	0.91		
San Antero	Bahía Cispata	-	77	-	6.41	3	525/224
				(IR: 4.35)	-		
				(Annual EIR: 30.31)	-		
Montelibano	Puerto Anchica	23	31	1.91	2.5	41/40	37/13
	Puerto Nuevo	10	30	0.83	-		

HBR: Human Biting Rate expressed as biting per person per night (b.p.n.). IR: Infection rate expressed in percentage. EIR: The annual entomological inoculation rate expressed as the number of infective bites per year.

*Specimens analyzed by ELISA were further tested by a second ELISA and a nested *Plasmodium* specific PCR. Number of specimens tested by ELISA/*Plasmodium* specific PCR (*n*/*n*.).

## Discussion

This study evaluated genetic population structure, geometric morphometrics and relevant entomological parameters for *An*. *nuneztovari* and *An*. *albimanus* at a microgeographic scale in the UCS malaria endemic region. A previous study of *An*. *nuneztovari* in Colombia showed that this species is unique, with a population subdivision between the west-northwest and the east-northeast, which suggested high genetic differentiation and reduction of gene flow among populations caused by distance, ecological differences and the presence of geographic barriers (i.e., the Andes) [20]. In the present study, a Bayesian genetic analysis using microsatellite markers, which exhibit a high mutation rate, allowed for the identification of population variation at a microgeographic scale. Of note, three co-occurrent *An*. *nuneztovari* subpopulations were detected that presented low genetic differentiation and high gene flow. In contrast, previous studies of *An*. *nuneztovari* in UCS using mitochondrial *COI* and nuclear *white* gene markers suggested genetic homogeneity in northwestern Colombia [21]. The present study used microsatellite markers which are characterized as codominant, highly polymorphic, having Mendelian inheritance, and are easily typed making them suitable genetic markers for analyzing population structure, genomic variation and evolutionary processes [61]. The present results reflect the utility of the microsatellite markers in detecting population variation in *An*. *nuneztovari*, showing higher sensitivity compared with mitochondrial and other nuclear markers. Similarly, a previous study on *An*. *nuneztovari* s.l. from the Brazilian Amazon region based on the same microsatellite markers, reported the presence of three genetic lineages [19]. Given our results in comparison with earlier data, we suggest that microsatellite markers are providing a more accurate picture of the current population structure of *An*. *nuneztovari* in UCS.

*Anopheles nuneztovari* geometric morphometric analysis revealed significant differences in size and wing conformation for several of the paired comparisons between populations. This species has shown high wing morphological variability previously in specimens from northwest Colombia [62, 63], Venezuela [64] and other regions of Latin America [65]. We found no relationship between the phenotypic and genetic distance matrix which indicates that the wing size differences detected among *An*. *nuneztovari* specimens are more likely associated with phenotypic plasticity rather than wing conformation variation as previously suggested for other anophelines [66]. It is also possible that wing size differences among populations are related to altitudinal differences among populations (0–76 m.a.s.l.). A previous study on *Anopheles cruzi* form São Paulo state in Brazil indicated vertical population structuring of wing geometry despite the similarity among landscape microenvironments; wing shapes were distinct between lowland and hilltop populations and the wings of hilltop specimens were larger [67].

For *An*. *albimanus* from UCS, Bayesian analysis detected two subpopulations with low genetic differentiation, high gene flow and no evidence of geographic differences. The absence of any obvious physical and biogeographic barriers in the UCS region may explain the low genetic differentiation recorded. Previous studies at a larger scale showed moderate to low genetic differentiation between Colombian Caribbean and Pacific populations of *An*. *albimanus* and suggested that this metapopulation was influenced by demographic events during the late Pleistocene [26, 27]. Furthermore, a panmictic population was detected between Central and South America [16, 24]. The Gómez et al [26] and Gutiérrez et al., [27] studies of Colombian *An*. *albimanus* were conducted with four microsatellite markers; in this study 11 microsatellites were evaluated and provided a finer resolution as shown by the Bayesian analysis that was able to identify three genetic subpopulations. We hypothesize that the low genetic differentiation and high gene flow in the present study is influenced by the dispersion capacity of *An*. *albimanus* adults, with an average flight range of 32 km [68]; in addition, the absence of a notable physical barrier such as high mountains or wide rivers in the endemic area likely favored gene flow. We hypothesize that adult mosquito movement by human or wind-assisted transport favors displacement of *An*. *albimanus* to nearby populations increasing gene flow and decreasing population structure.

Of note, significant variation in centroid size was observed in all paired comparisons for *An*. *albimanus* from Arboletes in the geometric morphometric analysis. Also, the multivariate alar conformation and genetic analysis showed a slight separation of the Arboletes population. A previous study of *An*. *albimanus* populations from the Colombia Caribbean and Pacific Coasts suggested size variation occurred as a consequence of environmental change experienced during the immature stages [26]. We hypothesize that in our study, Arboletes larval environments are distinctive in some essential parameters for anopheline development such as water temperature, *pH* and/or oxygen availability, as detected by Soleimani-Ahmadi et al. [69] in their study of anopheline larval habitat differences in southern Iran. In addition, factors such as food availability, larval population density and predators may influence intrapopulation adult size variation of Diptera [70].

In addition to genetic and phenetic evaluations, entomological indices were calculated because these estimates are fundamental tools for the design of malaria vector control strategies [71]; they provide information about changes in vector population dynamics that affect malaria transmission risk, which is important for determining when and where control measures are necessary. Variation in biting behavior has been reported for *An*. *nuneztovari* and *An*. *albimanus* across their Latin American distributions [28, 29, 72]. In this work, biting peaks for *An*. *nuneztovari* varied according to the locality; however, its main biting peaks in most localities were between 20:00–22:00 h. In other localities of this region, a similar biting peak for *An*. *nuneztovari* was observed [28, 30, 73]. Altogether, our results suggest the need to conduct regular surveys to monitor this vector behavior to appropriately direct control measures. For *An*. *albimanus*, biting peaks also varied, but the highest was found in Arboletes between 22:00–23:00 h. These values differ from those previously reported, in 2009, for this endemic area (*An*. *albimanus* 18:00–22:00 h) [73]; perhaps some environment parameters have been altered as a result of increasing anthropogenic activity in the region, influencing the biting time of this species. Variation in *An*. *nuneztovari* and *An*. *albimanus* biting behavior was not associated with genetic population differences, and several studies have demonstrated the effects of plasticity on *Anopheles* species biting behavior [74, 75]. Similar to the results of the present study, a recent investigation of *An*. *darlingi* from the Amazon region found an association of genetic diversity with biting behavior not related to population structure [76]. In addition, the variation in biting peaks for both vector species may be related with the degree of human exposure [28]. In this context, humans are under higher risk of being bitten by *Plasmodium* infected *Anopheles* when they carry out leisure activities in the open spaces of their houses, at the hours when the vectors show their higher biting activity.

In conclusion, results of entomological parameter estimations indicated that *An*. *nuneztovari* and *An*. *albimanus* exhibited variable biting behavior among localities and suggest that the UCS human populations are exposed continuously to malaria vector bites mainly during the early part of the night; therefore, control measures should focus to reduce vector-human contact at this time. In addition, the *Plasmodium* infection results for *An*. *nuneztovari* indicate that even the low human biting rate (< 2 b.p.n.) is sufficient to maintain malaria transmission, and although, *An*. *albimanus* is an important malaria vector, it was not detected infected; this can be due to the low infection rates usually detected in endemic regions of Colombia [28]. Furthermore, the low population structure detected for both vectors indicates high gene flow in the region, although local environmental characteristics may influence the wing conformation differentiation and behavioral variation. Our results indicate the importance of evaluating population structure and behavior at the local level to design the most cost-effective, targeted control strategies.

## Supporting information

S1 Table Microsatellite allele data derived from the *Anopheles nuneztovari* populations. (XLSX)

S2 Table Microsatellite allele data derived from the *Anopheles albimanus* populations. (XLSX)

S3 Table Paired estimates of genetic differentiation (*F*_*ST*_) and the number of migrants (*N*_*m*_) for the populations of *Anopheles nuneztovari*, in the endemic area Urabá-Bajo Cauca and Alto Sinú-Colombia. (DOCX)

S4 Table Paired estimates of genetic differentiation (*F*_*ST*_) and the number of migrants (*N*_*m*_) for the populations of *Anopheles albimanus*, in the endemic area Urabá-Bajo Cauca and Alto Sinú-Colombia. (DOCX)

S5 Table Wing centroid size comparison among populations of *Anopheles nuneztovari* from Urabá-Bajo Cauca and Alto Sinú. (DOCX)

S6 Table Wing centroid size comparison among populations of *Anopheles albimanus* from Urabá-Bajo Cauca and Alto Sinú. (DOCX)

S7 Table Relationship between the paired genetic structure (*F*_*ST*_), phenotypic differentiation (Mahalanobis distance), environmental distance (Circuitscape cost distance) and geographical distances among *Anopheles nuneztovari* populations in Urabá-Bajo Cauca and Alto Sinú. (DOCX)

S8 Table Relationship between the paired genetic structure (*F*_*ST*_), phenotypic differentiation (Mahalanobis distance), environmental distance (Circuitscape cost distance) and geographical distances between *Anopheles albimanus* populations in Urabá-Bajo Cauca and Alto Sinú. (DOCX)

## References

[1] WHO. World Health Organization (WHO) World Malaria Report 2020. Malaria report. Geneva: World Health Organization; 2020.

[2] Conn JE, Grillet ME, Correa M, Sallum MAM. Malaria Transmission in South America-Present Status and Prospects for Elimination. Towards Malaria Elimination—A Leap Forward. IntechOpen; 2018. doi: 10.5772/intechopen.76964

[3] INS. Instituto Nacional de Salud, Boletín epidemiológico Semanal. Estadísticas del sistema de vigilancia en salud pública- SIVIGILA, Casos totales en la Semana Epidemiológica 52 y acumulados del año, Subdirección de Vigilancia y Control en Salud Pública. 2020.

[4] INS. Instituto Nacional de Salud, Boletín epidemiológico Semanal. Estadísticas del sistema de vigilancia en salud pública- SIVIGILA, Casos totales en la Semana Epidemiológica 52 y acumulados del año, Subdirección de Vigilancia y Control en Salud Pública. 2019.

[5] INS. Instituto Nacional de Salud, Boletín epidemiológico Semanal. Estadísticas del sistema de vigilancia en salud pública- SIVIGILA, Casos totales en la Semana Epidemiológica 52 y acumulados del año, Subdirección de Vigilancia y Control en Salud Pública. 2018.

[6] INS. Instituto Nacional de Salud, Boletín epidemiológico Semanal. Estadísticas del sistema de vigilancia en salud pública- SIVIGILA, Casos totales en la Semana Epidemiológica 52 y acumulados del año, Subdirección de Vigilancia y Control en Salud Pública. 2017.

[7] INS. Instituto Nacional de Salud, Boletín epidemiológico Semanal. Estadísticas del sistema de vigilancia en salud pública- SIVIGILA, Casos totales en la Semana Epidemiológica 52 y acumulados del año, Subdirección de Vigilancia y Control en Salud Pública. 2016.

[8] INS. Instituto Nacional de Salud, Boletín epidemiológico Semanal. Estadísticas del sistema de vigilancia en salud pública- SIVIGILA, Casos totales en la Semana Epidemiológica 52 y acumulados del año, Subdirección de Vigilancia y Control en Salud Pública. 2013.

[9] INS. Instituto Nacional de Salud, Boletín epidemiológico Semanal. Estadísticas del sistema de vigilancia en salud pública- SIVIGILA, Casos totales en la Semana Epidemiológica 52 y acumulados del año, Subdirección de Vigilancia y Control en Salud Pública. 2014.

[10] Gonzalez R, Carrejo N. Introducción al estudio taxonómico de *Anopheles* de Colombia Claves y notas de distribución. 2nd ed. Cali: Universidad del Valle; 2009.

[11] Olano V, Brochero H, Sáenz R, Quiñones M, Molina J. Mapas preliminares de la distribución de especies de *Anopheles* vectores de malaria en Colombia. Biomédica. 2001;21: 402–408.

[12] Hernández-Valencia JC, Rincón DS, Marín A, Naranjo-Díaz N, Correa MM. Effect of land cover and landscape fragmentation on anopheline mosquito abundance and diversity in an important Colombian malaria endemic region. PLoS One. 2020;15. doi: 10.1371/journal.pone.0240207 33057442 PMC7561141

[13] McCoy KD. The population genetic structure of vectors and our understanding of disease epidemiology. Parasite, 2008;15: 444–448. doi: 10.1051/parasite/2008153444 18814720

[14] Ogola E, Villinger J, Mabuka D, Omondi D, Orindi B, Mutunga J, et al. Composition of *Anopheles* mosquitoes, their blood-meal hosts, and *Plasmodium falciparum* infection rates in three islands with disparate bed net coverage in Lake Victoria, Kenya. Malar J. 2017;16: 360–372. doi: 10.1186/s12936-017-2015-5 28886724 PMC5591540

[15] OPS. Estrategia para la toma de decisiones en el marco del manejo integrado de vectores de malaria (ED MIVM). Primera. 2013; 68. Available: http://www.paho.org/hq/index.php?option=com_docman&task=doc_download&gid=25778&Itemid=270&lang=es.

[16] Loaiza JR, Scott ME, Bermingham E, Sanjur OI, Wilkerson R, Rovira J, et al. Late Pleistocene environmental changes lead to unstable demography and population divergence of *Anopheles albimanus* in the northern Neotropics. Mol Phylogenet Evol. 2010;57: 1341–6. doi: 10.1016/j.ympev.2010.09.016 20888924 PMC3229172

[17] Scarpassa VM, Conn JE. Mitochondrial DNA detects a complex evolutionary history with Pleistocene Epoch divergence for the neotropical malaria vector *Anopheles nuneztovari* sensu lato. Am J Trop Med Hyg. 2011;85: 857–67. doi: 10.4269/ajtmh.2011.11–0150 22049039 PMC3205631

[18] Mirabello L, Conn JE. Population analysis using the nuclear white gene detects Pliocene/Pleistocene lineage divergence within *Anopheles nuneztovari* in South America. Med Vet Entomol. 2008;22: 109–19. doi: 10.1111/j.1365-2915.2008.00731.x 18498609

[19] Scarpassa VM, Cunha-Machado AS, Saraiva JF. Evidence of new species for malaria vector *Anopheles nuneztovari* sensu lato in the Brazilian Amazon region. Malar J. 2016;15: 205. doi: 10.1186/s12936-016-1217-6 27068120 PMC4828892

[20] Naranjo-Díaz N, Sallum MAM, Correa MM. Population dynamics of *Anopheles nuneztovari* in Colombia. Infect Genet Evol. 2016;45: 56–65. doi: 10.1016/j.meegid.2016.08.019 27553709

[21] Jaramillo LM, Gutiérrez LA, Luckhart S, Conn JE, Correa MM. Molecular evidence for a single taxon, *Anopheles nuneztovari* s.l., from two endemic malaria regions in Colombia. Mem Inst Oswaldo Cruz. 2011;106: 1017–23. doi: 10.1590/s0074-02762011000800020 22241127 PMC3321982

[22] De Merida AM, Palmieri M, Yurrita M, Molina A, Molina E, Black WC. Mitochondrial DNA variation among *Anopheles albimanus* populations. Am J Trop Med Hyg. 1999;61: 230–9. doi: 10.4269/ajtmh.1999.61.230 10463672

[23] Loaiza JR, Scott ME, Bermingham E, Rovira J, Conn JE. Evidence for pleistocene population divergence and expansion of *Anopheles albimanus* in Southern Central America. Am J Trop Med Hyg. 2010;82: 156–64. doi: 10.4269/ajtmh.2010.09–0423 20065014 PMC2803528

[24] Molina-Cruz A, de Mérida AMP, Mills K, Rodríguez F, Schoua C, Yurrita MM, et al. Gene flow among *Anopheles albimanus* populations in Central America, South America, and the Caribbean assessed by microsatellites and mitochondrial DNA. Am J Trop Med Hyg. 2004;71: 350–9. 15381818

[25] Gutiérrez LA, Naranjo N, Jaramillo LM, Muskus C, Luckhart S, Conn JE, et al. Natural infectivity of *Anopheles* species from the Pacific and Atlantic Regions of Colombia. Acta Trop. 2008;107: 99–105. doi: 10.1016/j.actatropica.2008.04.019 18554564

[26] Gómez GF, Márquez EJ, Gutiérrez LA, Conn JE, Correa MM. Geometric morphometric analysis of Colombian *Anopheles albimanus* (Diptera: Culicidae) reveals significant effect of environmental factors on wing traits and presence of a metapopulation. Acta Trop. 2014;135: 75–85. doi: 10.1016/j.actatropica.2014.03.020 24704285 PMC4464773

[27] Gutiérrez LA, Naranjo NJ, Cienfuegos AV, Muskus CE, Luckhart S, Conn JE, et al. Population structure analyses and demographic history of the malaria vector *Anopheles albimanus* from the Caribbean and the Pacific regions of Colombia. Malar J. 2009;8: 259. doi: 10.1186/1475-2875-8-259 19922672 PMC2789746

[28] Naranjo-Díaz N, Rosero DA, Rua-Uribe G, Luckhart S, Correa MM. Abundance, behavior and entomological inoculation rates of anthropophilic anophelines from a primary Colombian malaria endemic area. Parasit Vectors. 2013;6: 61. doi: 10.1186/1756-3305-6-61 23497535 PMC3637137

[29] Naranjo-Díaz N, Altamiranda M, Luckhart S, Conn JE, Correa MM. Malaria vectors in ecologically heterogeneous localities of the Colombian Pacific region. PLoS One. 2014;9: e103769. doi: 10.1371/journal.pone.0103769 25090233 PMC4121283

[30] Naranjo-Díaz N, Hernandez-Valencia JC, Marín A, Correa MM. Relationship between land cover and Anophelinae species abundance, composition and diversity in NW Colombia. Infect Genet Evol. 2020;78. doi: 10.1016/j.meegid.2019.104114 31707086

[31] Naranjo-Díaz N, Altamiranda-Saavedra M, Correa MM. *Anopheles* species composition and entomological parameters in malaria endemic localities of North West Colombia. Acta Trop. 2019;190: 13–21. doi: 10.1016/j.actatropica.2018.10.011 30367837

[32] Cienfuegos A V, Rosero DA, Naranjo N, Luckhart S, Conn JE, Correa MM. Evaluation of a PCR-RFLP-ITS2 assay for discrimination of *Anopheles* species in northern and western Colombia. Acta Trop. 2011;118: 128–35. doi: 10.1016/j.actatropica.2011.02.004 21345325 PMC3085589

[33] Cienfuegos A, Gómez G, Córdoba L, Luckhart S, Conn J, Correa M. Diseño y evaluación de metodologías basadas en PCR–RFLP de ITS2 para la identificación molecular de mosquitos *Anopheles* spp. (Diptera:Culicidae) de la Costa Pacífica de Colombia. Rev Biomed. 2008;19: 35–44.

[34] Zapata MA, Cienfuegos A V, Quirós OI, Quiñones ML, Luckhart S, Correa MM. Discrimination of seven *Anopheles* species from San Pedro de Uraba, Antioquia, Colombia, by polymerase chain reaction-restriction fragment length polymorphism analysis of its sequences. Am J Trop Med Hyg. 2007;77: 67–72. 17620632

[35] Folmer O, Black M, Hoeh W, Lutz R, Vrijenhoek R. DNA primers for amplification of mitochondrial cytochrome c oxidase subunit I from diverse metazoan invertebrates. Mol Mar Biol Biotechnol. 1994;3: 294–9. 7881515

[36] Gómez G, Jaramillo L, Correa MM. Wing geometric morphometrics and molecular assessment of members in the Albitarsis Complex from Colombia. Mol Ecol Resour. 2013;13: 1082–1092. doi: 10.1111/1755-0998.12126 23702155

[37] Olson D. M., Dinerstein E., Wikramanayake E. D., Burgess N. D., Powell G. V. N., Underwood E. C., et al. Terrestrial ecoregions of the world: a new map of life on Earth. Bioscience 51. 2001, (11):933–938.

[38] Penilla RP, Ranson H, Padilla N, Morgan JC, Steen K, Pignatelli P, et al. Towards a Genetic Map for *Anopheles albimanus*: Identification of Microsatellite Markers and a Preliminary Linkage Map for Chromosome 2. Am J Trop Med Hyg. 2009;81: 1007–1012. doi: 10.4269/ajtmh.2009.08–0607 19996429

[39] Glaubitz JC. Convert: A user-friendly program to reformat diploid genotypic data for commonly used population genetic software packages. Mol Ecol Notes. 2004;4: 309–310. doi: 10.1111/j.1471-8286.2004.00597.x

[40] Van Oosterhaut CH, Wills DPM, Shipley P. MICRO-CHECKER: software for identifying and correcting genotyping errors in microsatellite data. Mol Ecol Notes. 2004;4: 535–538.

[41] Falush D, Stephens M, Pritchard JK. Inference of population structure using multilocus genotype data: linked loci and correlated allele frequencies. Genetics. 2003;164: 1567–87. doi: 10.1093/genetics/164.4.1567 12930761 PMC1462648

[42] Kaddumukasa MA, Wright J, Muleba M, Stevenson JC, Norris DE, Coetzee M. Genetic differentiation and population structure of *Anopheles funestus* from Uganda and the southern African countries of Malawi, Mozambique, Zambia and Zimbabwe. Parasites and Vectors. 2020;13: 1–13. doi: 10.1186/s13071-020-3962-1 32070403 PMC7029513

[43] Earl DA, vonHoldt BM. STRUCTURE HARVESTER: a website and program for visualizing STRUCTURE output and implementing the Evanno method. Conserv Genet Resour. 2012;4: 359–361. doi: 10.1007/s12686-011-9548-7

[44] Jakobsson M, Rosenberg NA. CLUMPP: a cluster matching and permutation program for dealing with label switching and multimodality in analysis of population structure. Bioinformatics. 2007;23: 1801–1806. doi: 10.1093/bioinformatics/btm233 17485429

[45] Langella O. Populations 1.2.31. Population genetic software CNRS UPR9034. [accessed 2020, September, 8] Available from http://bioinformatics.org/~tryphon/populations.

[46] Lainhart W, Bickersmith SA, Nadler KJ, Moreno M, Saavedra MP, Chu VM, et al. Evidence for temporal population replacement and the signature of ecological adaptation in a major Neotropical malaria vector in Amazonian Peru. Malar J. 2015;14: 375. doi: 10.1186/s12936-015-0863-4 26415942 PMC4587789

[47] Belkhir K, Borsa P., Chikhi L, Raufaste N, Bonhomme F. GENETIX 4.05, logiciel sous Windows TM pour la génétique des populations. Laboratoire Génome, Populations, Interactions–ScienceOpen. Université de Montpellier II, Montpellier (France).; 2004. Available: https://www.scienceopen.com/document?vid=9a3e2cf3-2971-405c-8297-258227c3cb30.

[48] Excoffier L, Lischer HEL. Arlequin suite ver 3.5: a new series of programs to perform population genetics analyses under Linux and Windows. Mol Ecol Resour. 2010;10: 564–567. doi: 10.1111/j.1755-0998.2010.02847.x 21565059

[49] Srivastava A, Joshi SH, Mio W, Xiuwen Liu X. Statistical shape analysis: clustering, learning, and testing. IEEE Trans Pattern Anal Mach Intell. 2005;27: 590–602. doi: 10.1109/TPAMI.2005.86 15794163

[50] Bookstein FL. Morphometric tools for landmark data: geometry and biology. Cambridge University Press; 1997.

[51] Prudhomme J, Cassan C, Hide M, Toty C, Rahola N, Vergnes B, et al. Ecology and morphological variations in wings of *Phlebotomus ariasi* (Diptera: Psychodidae) in the region of Roquedur (Gard, France): a geometric morphometrics approach. Parasit Vectors. 2016;9: 578. doi: 10.1186/s13071-016-1872-z 27842606 PMC5109773

[52] Webster M, Sheets HD. A Practical Introduction to Landmark-Based Geometric Morphometrics. Paleontol Soc Pap. 2010;16: 163–188. doi: 10.1017/S1089332600001868

[53] Dujardin J-P. Morphometrics applied to medical entomology. Infect Genet Evol. 2008;8: 875–90. doi: 10.1016/j.meegid.2008.07.011 18832048

[54] Hammer Ø, Harper DAT a. T, Ryan PD. PAST: Paleontological Statistics Software Package for Education and Data Analysis. Palaeontol Electron. 2001;4: 1–9. doi: 10.1016/j.bcp.2008.05.025

[55] Guillot G, Mortier F, Estoup A. GENELAND: A computer package for landscape genetics. Mol Ecol Notes. 2005;5: 712–715. doi: 10.1111/j.1471-8286.2005.01031.x

[56] Phillips SJ, Anderson RP, Schapire RE. Maximum entropy modeling of species geographic distributions. Ecol Modell. 2006;190: 231–259.

[57] McRae BH, Beier P. Circuit theory predicts gene flow in plant and animal populations. Proc Natl Acad Sci. 2007;104: 19885–19890. doi: 10.1073/pnas.0706568104 18056641 PMC2148392

[58] Mantel N. The detection of disease clustering and a generalized regression approach. Cancer Res. 1967;27: 209–20. 6018555

[59] Singh B, Bobogare A, Cox-Singh J, Snounou G, Abdullah MS, Rahman HA: A genus- and species-specific nested polymerase chain reaction malaria detection assay for epidemiologic studies. Am J Trop Med Hyg. 1999;60:687–692. doi: 10.4269/ajtmh.1999.60.687 10348249

[60] da Silva-Vasconcelos A, Kató MYN, Mourão EN, de Souza RTL, Lacerda RN da L, Sibajev A, et al. Biting indices, host-seeking activity and natural infection rates of anopheline species in Boa Vista, Roraima, Brazil from 1996 to 1998. Mem Inst Oswaldo Cruz. 2002;97: 151–61. doi: 10.1590/s0074-02762002000200002 12016435

[61] Abdul-Muneer PM. Application of Microsatellite Markers in Conservation Genetics and Fisheries Management: Recent Advances in Population Structure Analysis and Conservation Strategies. Genet Res Int. 2014;2014: 1–11. doi: 10.1155/2014/691759 24808959 PMC3997932

[62] Calle L DA, Quiñones ML, Erazo HF, Jaramillo O N. Morphometric discrimination of females of five species of *Anopheles* of the subgenus *Nyssorhynchus* from southern and northwest Colombia. Mem Inst Oswaldo Cruz. 2002;97: 1191–5. 12563488 10.1590/s0074-02762002000800021

[63] Fajardo Ramos M, González Obando R, Fidel Suárez M, López D, Wilkerson R, Sallum MAM. Morphological analysis of three populations of *Anopheles (Nyssorhynchus) nuneztovari* Gabaldón (Diptera: Culicidae) from Colombia. Mem Inst Oswaldo Cruz. 2008;103: 85–92. 18368239 10.1590/s0074-02762008000100013

[64] Delgado N, Rubio-Palis Y. Morphometric characterization of the malaria vector *Anopheles nuñeztovari* (Diptera: Culicidae) from Western Venezuela. Mosq Syst. 1992;24: 231–241.

[65] Faran M. Mosquitos Studies (Díptera, Culicidae) XXXIV. A revision of the Albimanus Section of the subgenus *Nyssorhynchus* of *Anopheles*. Contrib Amer Ent Inst. 1980;15: 1–215.

[66] Ayala D, Caro-Riaño H, Dujardin J-P, Rahola N, Simard F, Fontenille D. Chromosomal and environmental determinants of morphometric variation in natural populations of the malaria vector *Anopheles funestus* in Cameroon. Infect Genet Evol. 2011;11: 940–7. doi: 10.1016/j.meegid.2011.03.003 21414420 PMC3665408

[67] Lorenz C, Marques TC, Sallum MAM, Suesdek L. Altitudinal population structure and microevolution of the malaria vector *Anopheles cruzii* (Diptera: Culicidae). Parasites and Vectors. 2014;7: 581. doi: 10.1186/s13071-014-0581-8 25511160 PMC4334843

[68] Frederickson E. Bionomía y control de *Anopheles albimanus*. Washigton, D.C.: Organizacion Panamericana de la Salud; 1993.

[69] Soleimani-Ahmadi M, Vatandoost H, Zare M. Characterization of larval habitats for anopheline mosquitoes in a malarious area under elimination program in the southeast of Iran. Asian Pac J Trop Biomed. 2014;4: S73–80. doi: 10.12980/APJTB.4.2014C899 25183151 PMC4025279

[70] Paaijmans KP, Huijben S, Githeko AK, Takken W. Competitive interactions between larvae of the malaria mosquitoes *Anopheles arabiensis* and *Anopheles gambiae* under semi-field conditions in western Kenya. Acta Trop. 2009;109: 124–130. doi: 10.1016/j.actatropica.2008.07.010 18760989

[71] Rubio-Palis Y, Bevilacqua M, Medina DA, Moreno JE, Cárdenas L, Sánchez V, et al. Malaria entomological risk factors in relation to land cover in the Lower Caura River Basin, Venezuela. Mem Inst Oswaldo Cruz. 2013;108: 220–8. doi: 10.1590/0074-0276108022013015 23579803 PMC3970659

[72] Galardo AKR, Arruda M, D’Almeida Couto A A R, Wirtz R, Lounibos LP, Zimmerman RH. Malaria vector incrimination in three rural riverine villages in the Brazilian Amazon. Am J Trop Med Hyg. 2007;76: 461–9. 17360868

[73] Gutiérrez LA, González JJ, Gómez GF, Castro MI, Rosero DA, Luckhart S, et al. Species composition and natural infectivity of anthropophilic *Anopheles* (Diptera: Culicidae) in the states of Córdoba and Antioquia, Northwestern Colombia. Mem Inst Oswaldo Cruz. 2009;104: 1117–24. 20140372 10.1590/s0074-02762009000800008 PMC3066193

[74] Ryan SJ, Lippi CA, Boersch-Supan PH, Heydari N, Silva M, Adrian J, et al. Quantifying seasonal and diel variation in Anopheline and Culex human biting rates in southern Ecuador. Malar J. 2017;16: 479. doi: 10.1186/s12936-017-2121-4 29166907 PMC5700746

[75] Maliti DV, Marsden CD, Main BJ, Govella NJ, Yamasaki Y, Collier TC, et al. Investigating associations between biting time in the malaria vector *Anopheles arabiensis* Patton and single nucleotide polymorphisms in circadian clock genes: Support for sub-structure among *An*. *arabiensis* in the Kilombero valley of Tanzania. Parasites and Vectors. 2016;9: 1–15. doi: 10.1186/S13071-016-1394-8 26920563 PMC4769569

[76] Campos M, Alonso DP, Conn JE, Vinetz JM, Emerson KJ, Ribolla PEM. Genetic diversity of *Nyssorhynchus (Anopheles) darlingi* related to biting behavior in western Amazon. Parasites and Vectors. 2019;12: 1–9. doi: 10.1186/S13071-019-3498-4 31101131 PMC6525393

