# Supplementary material for: Entomological parameters and population structure at a microgeographic scale of the main Colombian malaria vectors Anopheles albimanus and Anopheles nuneztovari
Source: PLoS One. 2023 Jan 6;18(1):e0280066. doi: 10.1371/journal.pone.0280066 (PMC9821454; doi:10.1371/journal.pone.0280066)
Supplement: S3 Table — (DOCX) [file pone.0280066.s003.docx]

**S3 Table.** Paired estimates of genetic differentiation (*F_ST_*) and the number of migrants (*N_m_*) for the populations of *Anopheles nuneztovari*, in the endemic area Urabá-Bajo Cauca and Alto Sinú-Colombia.

| **Population** | **El Bagre** | **Mutatá** | **Cáceres** | **Tierralta** | **Turbo** |
| --- | --- | --- | --- | --- | --- |
| **El Bagre** | _____ | 11.1 | 26.9 | 12.7 | 12.6 |
| **Mutatá** | 0.0431 * | _____ | 12.9 | 13.7 | 22.8 |
| **Cáceres** | 0.0182 | 0.0372 * | _____ | 64.5 | 21.5 |
| **Tierralta** | 0.0378 * | 0.0351 * | 0.0076 | _____ | 34.7 |
| **Turbo** | 0.0380 * | 0.0214 * | 0.0226 | 0.0142 | _____ |

Above the diagonal Nm values ​​and below the diagonal *F_ST_* values. * Indicates statistical significance after Bonferroni sequential correction, *p* <0.05.
