# Supplementary material for: Entomological parameters and population structure at a microgeographic scale of the main Colombian malaria vectors Anopheles albimanus and Anopheles nuneztovari
Source: PLoS One. 2023 Jan 6;18(1):e0280066. doi: 10.1371/journal.pone.0280066 (PMC9821454; doi:10.1371/journal.pone.0280066)
Supplement: S4 Table — (DOCX) [file pone.0280066.s004.docx]

**S4 Table**. Paired estimates of genetic differentiation (*F_ST_*) and the number of migrants (*N_m_*) for the populations of *Anopheles albimanus*, in the endemic area Urabá-Bajo Cauca and Alto Sinú-Colombia.

| **Populations** | **Arboletes** | **Montelibano** | **Moñitos** | **San Antero** | **Turbo** |
| --- | --- | --- | --- | --- | --- |
| **Arboletes** | _____ | 38.1 | 132.3 | 53.3 | 42.4 |
| **Montelibano** | 0.0129 | _____ | 30.3 | 17.9 | 30.7 |
| **Moñitos** | 0.0037 | 0.0161 | _____ | 526.4 | 64.8 |
| **San Antero** | 0.0092 | 0.2712 * | 0.0009 | _____ | 166.5 |
| **Turbo** | 0.0116 | 0.016 * | 0.0076 | 0.0029 | _____ |

Above the diagonal Nm values ​​and below the diagonal *F_ST_* values. * Indicates statistical significance after Bonferroni sequential correction, *p* <0.05.
