# Supplementary material for: Entomological parameters and population structure at a microgeographic scale of the main Colombian malaria vectors Anopheles albimanus and Anopheles nuneztovari
Source: PLoS One. 2023 Jan 6;18(1):e0280066. doi: 10.1371/journal.pone.0280066 (PMC9821454; doi:10.1371/journal.pone.0280066)
Supplement: S5 Table — (DOCX) [file pone.0280066.s005.docx]

**S5 Table.** Wing centroid size comparison among populations of *Anopheles nuneztovari* from Urabá-Bajo Cauca and Alto Sinú.

| **Populations** | **Cáceres** | **Tierralta** | **Turbo** | **Mutatá** | **El Bagre** |
| --- | --- | --- | --- | --- | --- |
| **Cáceres** | ___ | 0.86 | 0.33 | 0.11 | 0.07 |
| **Tierralta** | 0.5 | ___ | 0.46 | 0.2 | 0.07 |
| **Turbo** | 0.74 | 0.33 | ___ | 0.6 | 0.004 * |
| **Mutatá** | 0.24 | 0.06 | 0.41 | ___ | 0.002 * |
| **El Bagre** | 0.16 | 0.04 * | 0.29 | 0.81 | ___ |

Comparison of *p*-values ​​of means (above the diagonal) and variances (below the diagonal). * Indicates statistical significance after Bonferroni sequential correction, *p* <0.05.
