# Supplementary material for: Entomological parameters and population structure at a microgeographic scale of the main Colombian malaria vectors Anopheles albimanus and Anopheles nuneztovari
Source: PLoS One. 2023 Jan 6;18(1):e0280066. doi: 10.1371/journal.pone.0280066 (PMC9821454; doi:10.1371/journal.pone.0280066)
Supplement: S6 Table — (DOCX) [file pone.0280066.s006.docx]

**S6 Table.** Wing centroid size comparison among populations of *Anopheles albimanus* from Urabá-Bajo Cauca and Alto Sinú.

| **Populations** | **Arboletes** | **Moñitos** | **San Antero** | **Turbo** | **Montelibano** |
| --- | --- | --- | --- | --- | --- |
| **Arboletes** |  | 0.23 | 0.61 | 0.8 | 0.2 |
| **Moñitos** | 0.002 * |  | 0.4 | 0.04 * | 0.85 |
| **San Antero** | 0.04 * | 0.27 |  | 0.3 | 0.31 |
| **Turbo** | 0.002 * | 0.97 | 0.28 |  | 0.05 |
| **Montelibano** | 0.0002 * | 0.35 | 0.05 | 0.34 |  |

Comparison of *p*-values ​​of means (above the diagonal) and variances (below the diagonal). * Indicates statistical significance after Bonferroni sequential correction, *p* <0.05.
