# Supplementary material for: Entomological parameters and population structure at a microgeographic scale of the main Colombian malaria vectors Anopheles albimanus and Anopheles nuneztovari
Source: PLoS One. 2023 Jan 6;18(1):e0280066. doi: 10.1371/journal.pone.0280066 (PMC9821454; doi:10.1371/journal.pone.0280066)
Supplement: S7 Table — (DOCX) [file pone.0280066.s007.docx]

**S7 Table.** Relationship between the paired genetic structure (*F_ST_*), phenotypic differentiation (Mahalanobis distance), environmental distance (Circuitscape cost distance) and geographical distances among *Anopheles nuneztovari* populations in Urabá-Bajo Cauca and Alto Sinú.

| **Distance matrices** | *r* | *p* |
| --- | --- | --- |
| Genetic + Geographic | 0.71 | 0.02 * |
| Genetic + Environmental | -0.41 | 0.22 |
| Phenotypic + Geographic | -0.25 | 0.42 |
| Phenotypic + Environmental | 0.28 | 0.47 |
| Phenotypic + Genetic | -0.34 | 0.33 |

* Indicates statistical significance after Bonferroni sequential correction, *p* <0.05.
