# Supplementary material for: Entomological parameters and population structure at a microgeographic scale of the main Colombian malaria vectors Anopheles albimanus and Anopheles nuneztovari
Source: PLoS One. 2023 Jan 6;18(1):e0280066. doi: 10.1371/journal.pone.0280066 (PMC9821454; doi:10.1371/journal.pone.0280066)
Supplement: S8 Table — (DOCX) [file pone.0280066.s008.docx]

**S8 Table.** Relationship between the paired genetic structure (*F_ST_*), phenotypic differentiation (Mahalanobis distance), environmental distance (Circuitscape cost distance) and geographical distances between *Anopheles albimanus* populations in Urabá-Bajo Cauca and Alto Sinú.

| **Distance matrices** | *r* | *p* |
| --- | --- | --- |
| Genetics + Geographic | 0.44 | 0.26 |
| Genetics + Environmental | 0.23 | 0.68 |
| Phenotypic + Geographic | -0.55 | 0.09 |
| Phenotypic + Environmental | 0.42 | 0.22 |
| Phenotypic + Genetics | 0.22 | 0.5 |

* Indicates statistical significance after Bonferroni sequential correction, *p* <0.05.
